# In Vitro Functional Characterization of the −143 A>C Variant in the Porcine *DLD* Promoter and Its Modulation of KLF2-Dependent Repression and Myoblast Phenotypes

**DOI:** 10.3390/ani16213391

**Published:** 2026-10-27

**Authors:** Mengke Song, Rongru Zhu, Wentao Wang, Yuanlu Sun, Xiaomei Ma, Siqi Yang, Ming Tian, Xinmiao He, Bohan Rong, Xiuqin Yang

**Affiliations:** 1 College of Animal Science and Technology, Northeast Agricultural University, Harbin 150030, China; 2 Institute of Animal Husbandry, Heilongjiang Academy of Agricultural Sciences, No. 368 Xuefu Road, Nangang District, Harbin 150086, China

**Keywords:** DLD, SNP, KLF2, myoblasts proliferation, subtype-specific myogenic differentiation

## Abstract

Meat quality in pigs is fundamentally shaped by the relative abundance and metabolic properties of distinct skeletal muscle fiber types. Dihydrolipoyl dehydrogenase (DLDH), an indispensable catalytic subunit of mitochondrial dehydrogenase complexes, has been documented to link with myocyte development; nevertheless, the effects of the causal regulatory variants governing transcription of its encoding gene *DLD* on the myogenic fate remain largely uncharacterized. In this study, comparative polymorphism analysis between Large White and Min pigs uncovered a regulatory single nucleotide polymorphism (SNP) at position −143 A>C within the porcine *DLD* promoter. Mechanistic dissection using dual-luciferase reporter, electrophoretic mobility shift, and chromatin immunoprecipitation assays revealed that the C allele strengthens Krüppel-like factor 2 (KLF2) DNA-binding affinity and potentiates KLF2-mediated transcriptional repression, leading to significantly reduced *DLD* expression. Functional characterization in porcine primary myoblasts demonstrated that *DLD* promotes myoblast proliferation by accelerating G2/M transition and upregulating cell-cycle regulators while suppressing terminal differentiation. Furthermore, *DLD* overexpression inhibited slow-twitch (myosin heavy chain (MYH) 7-positive) myogenic differentiation and suppressed the expression of myogenic regulatory factors *MYOD* and *MYOG*, whereas *DLD* knockdown produced converse effects. Taken together, these multiple lines of evidence suggest that the −143 A>C variant may reinforce transcriptional repression of *DLD* by increasing KLF2-binding occupancy. Because reduced *DLD* expression favors MYH7-positive myotube formation in our myoblast assays, this variant is hypothesized to influence myotube subtype balance. These findings may provide a potential molecular mechanism contributing to pork-quality variation and suggest the −143 A>C variant on the *DLD* promoter as a candidate marker for future genomic-selection studies.

## Simple Summary

Pork taste and tenderness are largely decided by different muscle-fiber types inside pig muscle. Current evidence indicates that pork containing more slow-twitch muscle fibers tends to deliver better-quality meat within reasonable physiological limits. While Chinese indigenous Min pigs generally yield superior pork quality relative to commercial Large White pigs, the genetic variants underlying this phenotypic disparity remain incompletely resolved. In this work, we compared gene sequences between these two pig breeds and found a single-base genetic change located in the promoter region of the *DLD* gene. When this genetic variant carries the C-form, it attracts more of the KLF2 regulatory protein, which reduces *DLD* gene activity. Cell tests showed that higher *DLD* expression speeds up muscle-stem-cell multiplication and stops these cells from developing into slow-twitch muscle fibers. By contrast, blocked *DLD* expression promotes the formation of slow-twitch muscle fibers linked to better-quality pork. This work provides a molecular-mechanistic dissection of a naturally occurring promoter variant of the *DLD* gene that modulates myogenic differentiation. Notably, this SNP locus may hold potential as a candidate marker for assisted selection to improve pork quality traits, offering a plausible molecular basis for the superior meat quality of Min pigs.

## 1. Introduction

Key metrics for evaluating pork meat quality include meat color, tenderness, water-holding capacity, intramuscular fat (marbling), post-mortem pH, and flavor [1]. Embryonic myogenesis and the terminal differentiation of skeletal muscle cells into distinct slow- and fast-twitch fiber subtypes constitute the cellular basis for inter-individual/varietal variation in these traits [2,3]. Slow-twitch type I fibers express myosin heavy chain (MYH) 7, whereas fast-twitch fibers comprise three subtypes: type IIa (MYH2), IIx (MYH1), and IIb (MYH4), each defined by its respective MYH isoform [2,4]. The distinct structural and metabolic signatures of these fiber subtypes directly shape the physicochemical properties of pork [3,5]. Slow-twitch type I fibers predominantly rely on oxidative metabolism, characterized by abundant mitochondria and myoglobin as well as low glycogen reserves [6]. Owing to their limited glycogen reserves and consequently reduced post-mortem glycolysis, type I fibers exhibit a higher ultimate pH, darker red color, and superior water-holding capacity [6,7,8]. Such fibers are also associated with greater intermyofibrillar lipid content (including triglycerides and phospholipids), collectively conferring improved tenderness, juiciness, and flavor [9]. By contrast, type IIb fast-twitch fibers operate primarily via glycolytic metabolism, are characterized by larger fiber diameter and lower myoglobin abundance, and possess high glycogen reserves [9]. Rapid glycogen catabolism post-mortem triggers accelerated pH decline while carcass temperature remains elevated, leading to pale, soft, and exudative meat, a major quality defect historically associated with commercial lean pig lines [7,9]. Consequently, identifying the genetic loci and regulatory variants that govern myogenic lineage commitment and fiber-type specification holds substantial promise for advancing marker-assisted and genomic selection strategies in pig breeding programs.

Dihydrolipoyl dehydrogenase (DLDH) represents an evolutionarily conserved flavoprotein dehydrogenase [10,11]. As an indispensable catalytic subunit (E3 component) of core mitochondrial enzyme assemblies including the pyruvate dehydrogenase complex (PDC), α-ketoglutarate dehydrogenase complex, and branched-chain α-keto acid dehydrogenase complex, DLDH is essential for the reoxidation of dihydrolipoyl moieties and reduced nicotinamide adenine dinucleotide generation, thereby sustaining mitochondrial energy metabolism and cellular redox homeostasis [10,11]. Notably, increasing advances in the understanding of myogenic differentiation, characterized by extensive mitochondrial remodeling and a shift in redox homeostasis, have challenged the traditional view of DLDH as solely a metabolic enzyme [12]. Instead, it has been considered an emerging target in the fields of myocyte development and muscle tissue regeneration [13]. However, current research has predominantly focused on the coding region of the *DLD* gene, leveraging gene-editing technologies to construct knockouts in animal models [14,15,16,17]. By contrast, genetic dissection of non-coding regulatory regions remains notably sparse. Especially, effector single nucleotide polymorphisms (SNPs) within these regulatory domains lack systematic investigation, and the molecular mechanisms that cis-regulate DLD transcriptional efficiency and influence myocyte differentiation remain poorly defined. Given the distinct myofiber composition across skeletal muscle depots, unravelling the regulatory basis of *DLD* expression disparities carries tangible implications for the genetic improvement of pork quality.

Herein, we identified a regulatory SNP at position −143 (A>C) in the porcine *DLD* promoter that strengthens KLF2 DNA-binding affinity and potentiates KLF2-mediated transcriptional repression, thereby reducing basal *DLD* expression. Functional assays further demonstrated that *DLD* promotes myoblast proliferation while suppressing terminal differentiation, and that enhanced *DLD* expression suppresses terminal differentiation and slow-twitch myogenic commitment. These findings suggest a potential link between porcine *DLD* promoter genetic variation, transcription factor occupancy, and myofiber type determination and provide a mechanistic framework that may inform future investigations into the genetic determinants of muscle fiber composition and pork quality.

## 2. Materials and Methods

### 2.1. Animals, Samples, and Nucleic Acid Isolation

*Longissimus dorsi* muscle samples were collected from 39 individuals (19 Large White pigs and 20 Min pigs, 22 females and 17 castrated males, 210-day-old) at the Institute of Animal Husbandry, Heilongjiang Academy of Agricultural Sciences, Harbin, China, for total RNA isolation. Ear tissue biopsies were collected from these same 39 animals for genomic DNA extraction used for promoter polymorphism analysis. These tissue samples were washed and treated with phosphate-buffered saline (PBS) and then placed in sterilized cryostorage tubes. They were then quickly placed in a liquid nitrogen tank for rapid freezing and returned to the laboratory for storage at −80 °C. Total RNA was isolated with TRIzol reagent (Invitrogen, Carlsbad, CA, USA). Genomic DNA was isolated using the standard phenol-chloroform method without modification [18]. All animal experiments were approved by and performed in strict accordance with the guidelines of the Animal Care Committee of Northeast Agricultural University.

### 2.2. Polymorphism Analysis

For in vivo *DLD* expression assay in *longissimus dorsi* muscle, three AA and three CC animals were selected from the same breed (Min) to exclude breed confounding factors. The promoter fragment of the *DLD* gene (Gene ID: 397129) ranging from −350 to +60 bp was amplified with primers shown in Table S1, and the products were sequenced directly by Genesoul Technology (Harbin, China). The sequences were aligned with SnapGene (version 6.0.2) to screen for SNPs. Candidate SNP sites were confirmed by manual inspection of the sequencing chromatograms using Chromas software (Version 2.6.6; https://technelysium.com.au/wp/chromas/, accessed 10 March 2021), which also determined the genotype of each animal.

### 2.3. Plasmids and Small-Interfering RNA (siRNA)

For construction of wild-type (WT) reporter vectors, the upstream sequences of porcine *DLD* spanning from −350 to +60 bp were then amplified and inserted into the SmaI site of the pGL3-BASIC vector. Meanwhile, targeted mutations were introduced into the promoter sequence of the *DLD* gene via overlap extension PCR to obtain mutant-type (MT) promoter fragments, which were subsequently inserted into the pGL3-BASIC vector to construct MT reporter vectors. All recombinant vectors were sequenced and verified by Genesoul Technology. Reporter constructs in treatment groups were generated separately according to the mutant base types at the five identified SNP loci (named −72, −98, −143, −276, −303).

The full-length coding sequences (CDSs) of porcine *DLD* and *KLF2* were subcloned into the pCMV-HA-C vector (encoding a C-terminal HA tag) to construct eukaryotic expression vectors at the *Kpn*I site using the ClonExpress II One Step Cloning Kit (Vazyme, Nanjing, China). siRNAs targeting *DLD* were synthesized by General Biol (Hefei, China) and their sequences are listed in Table S1.

### 2.4. Transcription Factor Prediction

The online prediction databases, including Promo (https://alggen.lsi.upc.es/cgi-bin/promo_v3/promo/promoinit.cgi?dirDB=TF_8.3, accessed 10 March 2021), AnimalTFDB 4.0 (https://guolab.wchscu.cn/AnimalTFDB4/, accessed 10 March 2021), and JASPAR (https://jaspar.genereg.net/, accessed 10 March 2021), were used to search for candidate transcription factors whose binding might be disrupted by specific SNPs of the *DLD* promoter.

### 2.5. Porcine Primary Myoblasts Isolation

*Longissimus dorsi* muscle tissues were harvested from 1-day-old Min piglets. Tissues were washed three times with PBS supplemented with 100 U/mL penicillin and 100 µg/mL streptomycin, followed by careful dissection to remove visible blood vessels, adipose tissue, and connective tissue. The muscle tissue was minced into approximately 1-mm^3^ pieces and digested with 1 mg/mL collagenase I (Biofroxx, Eichgraben, Bavaria, Germany) in high-glucose Dulbecco’s Modified Eagle’s Medium (DMEM, Gibco, Grand Island, NY, USA) at 37 °C for 1 h with gentle shaking. The partially digested tissue was further dissociated with 0.25% trypsin-EDTA at 37 °C for 30 min. The digestion was terminated by adding an equal volume of growth medium, and the cell suspension was successively filtered through 100 µm and 70 µm cell strainers. After centrifugation (800× *g*, 10 min), the cell pellet was resuspended in growth medium and pre-plated on uncoated 15 cm dishes for 1.5 h at 37 °C in 5% CO_2_ to allow fibroblasts to adhere. The non-adherent cells were collected, centrifuged, and seeded onto dishes pre-coated with poly-L-lysine (100 µg/mL) and fibronectin (10 µg/mL) (Sigma-Aldrich, St. Louis, MO, USA), and cultured in growth medium supplemented with 10 ng/mL bFGF at 37 °C in 5% CO_2_ until reaching 50–70% confluence.

For the following functional assays, three independent biological replicates refer to primary myoblasts isolated from three separate 1-day-old Min piglets (*n* = 3 animals). For each biological replicate, cell isolation, culture, transfection (or transduction), and data acquisition were performed independently. Within each biological replicate, three technical replicates (parallel culture wells derived from the same transfection batch) were included for each readout, and technical replicate values were averaged to generate a single data point per biological replicate for subsequent statistical analysis. All assays were performed using cells at passages 2–3.

### 2.6. Cell Culture, Transfection and Differentiation

Porcine myoblasts were maintained in high-glucose DMEM (Gibco) supplemented with 20% FBS, 1% penicillin–streptomycin, and 10 ng/mL basic fibroblast growth factor (Gibco). PK-15 cells were cultured in high-glucose DMEM (SEVEN Bio, Beijing, China) containing 10% FBS, 1% penicillin–streptomycin, and 1× non-essential amino acids (NEAA, Gibco).

For transient transfection assays, porcine myoblasts were transfected using Lipofectamine 3000 (Invitrogen, Carlsbad, CA, USA). Briefly, 0.5 µg of plasmid DNA and 1 µL of P3000 reagent were diluted in 25 µL of Opti-MEM (Gibco), mixed with 1.5 µL of Lipofectamine 3000 diluted in 25 µL of Opti-MEM, incubated for 15 min at room temperature, and added to cells. For siRNA-mediated knockdown, myoblasts were transfected with 100 nM siRNA per well; siRNA and Lipofectamine 3000 were diluted in Opti-MEM according to the manufacturer’s protocol, using the same total reaction volume as for plasmid transfection. PK-15 cells were transfected with Lip8000 transfection reagent (Beyotime, Shanghai, China) following the manufacturer’s standard protocols. In transfection experiments, porcine myoblasts were seeded at a density of 1.5 × 10^4^ cells per well in 24-well plates and transfected when cell confluence reached 70–80%. At 24 h post-transfection, growth medium was replaced with differentiation medium consisting of high-glucose DMEM (Gibco), 2% horse serum (Gibco), and 1% penicillin–streptomycin. Overexpression and knockdown efficacy were functionally validated by qRT-PCR and Western blot assays for target-gene expression in each biological replicate.

For PK-15 and 293T cell-line experiments, three independent biological replicates refer to fully independent experimental runs with separate cell seeding, transfection and sample collection, initiated from distinct low-passage cell aliquots.

### 2.7. Dual-Luciferase Reporter Assay

PK-15 cells were seeded in 24-well plates and cultured until the cell confluence reached 65–70%. Each reporter vector was transiently co-transfected into PK-15 cells with the Renilla luciferase reference plasmid *pRL-TK*, either alone or together with the *KLF2* overexpression vector, using the Lipo8000 Transfection Reagent (Beyotime, Shanghai, China). Luciferase activity was determined as previously described by a dual-luciferase reporter gene assay kit (Beyotime, Shanghai, China) after transfection for 48 h [18]. The relative luciferase activity was calculated as a ratio of firefly to Renilla luciferase values.

### 2.8. Western Blotting

At 48 h post-transfection, cells were collected for protein isolation using RIPA buffer (Beyotime, Shanghai, China) containing a protease inhibitor (Beyotime, Shanghai, China). A total of 10~25 µg of protein was loaded onto an SDS-polyacrylamide gel and electrophoresed on a 5% concentrated gel at 90 V for 30 min, followed by separation at 120 V for 90 min. The proteins were then transferred to a polyvinyl difluoride membrane (Millipore, Shanghai, China). After blocking with 5% skimmed milk at 37 °C for 2 h, membranes were incubated at 4 °C overnight with anti-HA tag (Mouse, 1:5000 dilution; AntibodySystem, Shanghai, China; Cat#: HY-P8094BA), anti- proliferating cell nuclear antigen (PCNA, Mouse, 1:5000 dilution; Proteintech, Shanghai, China; Cat#: 60097-1-Ig), anti-β-tubulin (Mouse, 1:20,000 dilution; Abiowell, Changsha, Hunan, China; Cat#: AWA86003), anti-β-actin (Rabbit, 1:20,000 dilution; Abiowell, Changsha, hunan, China; Cat#: AWA80113) primary antibodies. β-actin was used as a control in the experiments. Protein bands were visualized using the Odyssey^®^ DLx near-infrared imaging system (LI-COR Biosciences, Lincoln, NE, USA). Gray value analysis was conducted by ImageJ software (version 5.2.5) to calculate relative intensities of the bands.

### 2.9. Quantitative Real-Time PCR

Total RNA was extracted from *longissimus dorsi* muscle tissues using TRIzol reagent (Invitrogen, Carlsbad, CA, USA) according to the manufacturer’s instructions, and 500 ng of total RNA was reverse transcribed into cDNA using the HiScript III 1st Strand cDNA Synthesis Kit with gDNA wiper (Vazyme, Nanjing, China), as previously described [18]. Primers were designed in accordance with the porcine sequence (Table S1) and sent to Genesoul Technology (Harbin, China) for synthesis. Quantitative PCR was performed in a total volume of 10 μL containing 1× SYBR Premix (Vazyme Biotech, Nanjing, China), 0.2 μM each primer, and 1 μL cDNA. Amplification was performed on the StepOnePlus™ Real-Time PCR System (Applied Biosystems, Foster City, CA, USA) under the following conditions: 95 °C for 30 s, followed by 40 cycles of 95 °C for 5 s and 60 °C for 30 s, with a final melt-curve step. mRNA levels were normalized to *β-actin* as the internal reference, and relative expression levels were calculated using the 2^−ΔΔCt^ method. All reactions were performed in triplicate.

### 2.10. Electrophoretic Mobility Shift Assay

Nuclear extracts were isolated from 293T cells transfected with the KLF2-overexpressing plasmid using the nuclear extraction kit (Solarbio, Beijing, China). Electrophoretic mobility shift assay (EMSA) was performed with a chemiluminescent kit (Beyotime, Shanghai, China) according to the manufacturer’s instructions. The probes, including biotin-labeled, unlabeled, and mutant ones for *DLD*-specific binding, were synthesized by General Biol (Hefei, China), and the sequences are listed in Table S1. After being annealed to double-stranded oligonucleotides, the probes were incubated with 20 µg nuclear extracts for 20 min at room temperature. In the competition group, unlabeled oligonucleotides were first incubated for 10 min before the addition of labelled probe. The mixture was electrophoresed on a 5% polyacrylamide gel at 100 V for 1 h, and then transferred to a positively charged nylon membrane (Beyotime, Shanghai, China). The gels were visualized on an Azure c300 Gel Imaging System (Azure Biosystems, Dublin, CA, USA).

### 2.11. Chromatin Immunoprecipitation-Real-Time Quantitative PCR (ChIP-qPCR)

ChIP-qPCR was performed in PK-15 cells using the SimpleChIP^®^ Enzymatic Chromatin IP Kit (Cell Signaling Technology, Danvers, MA, USA) according to the manufacturer’s instructions. PK-15 cells were transfected with the *KLF2*-overexpressing plasmid. At 48 h post-transfection, cells were cross-linked with 1% formaldehyde (diluted from 37% stock) at room temperature for 10 min, and the reaction was quenched by adding glycine to a final concentration of 0.125 M for 5 min. Cells were washed twice with ice-cold PBS, scraped, and collected by centrifugation at 2000× *g* for 5 min at 4 °C. The cell pellets were resuspended in Buffer A containing 1× protease inhibitor cocktail, and chromatin was digested with micrococcal nuclease (0.5 µL per 4 × 10^6^ cells) at 37 °C for 20 min. The digestion was terminated by adding EDTA (final concentration 10 mM) and incubating on ice for 1–2 min. After centrifugation at 2000× *g* for 1 min at 4 °C, the nuclear pellets were resuspended in Buffer B and sonicated to release chromatin, yielding DNA fragments of approximately 150–900 bp (verified by agarose gel electrophoresis). The chromatin solution was incubated overnight at 4 °C under gentle rotation with an anti-HA tag antibody (Mouse, 1:5000 dilution; AntibodySystem, Shanghai, China; Cat#: HY-P8094BA) or normal IgG as a negative control. Immunocomplexes were captured with Protein G magnetic beads, washed, and eluted, and cross-links were reversed at 65 °C, followed by DNA purification. Immunoprecipitated and input DNA were subjected to qPCR with primers listed in Table S1, and the enrichment was calculated as the percentage of input (% input).

### 2.12. Cell Counting Kit-8 (CCK-8) Assay

Porcine myoblasts were transiently transfected with pCMV-HA-*DLD* or pCMV-HA (empty vector) plasmid, or with negative control (NC) or *DLD*-targeted siRNA (si-220) for 24 h and further cultured until the CCK-8 assay was performed. In CCK-8 assays, cells were incubated with 10% CCK-8 (Beyotime, Shanghai, China) in complete medium for 2 h at 37 °C. The optical density (OD) at 450 nm was measured using a Tecan Infinite F50 microplate reader (Tecan, Männedorf, Switzerland).

### 2.13. Flow Cytometry

Porcine myoblasts were inoculated in six-well plates at a density of 1 × 10^6^ cells per well and cultured for 24 h. Cells were transfected with various vectors (including pCMV-HA-*DLD*, empty vector, si-*220*, or NC) and cultured for another 24 h. Cells were harvested by trypsinization, washed twice with PBS, and fixed in ice-cold 70% ethanol at −20 °C overnight. After washing with PBS, cells were stained with the Cell Cycle Staining Kit (MultiSciences, Hangzhou, China) at 37 °C for 30 min in the dark. Cell cycle distribution was analyzed using a FACSCalibur flow cytometer (BD Biosciences, San Jose, CA, USA), and the percentages of cells in G0/G1, S, and G2/M phases were determined using ModFit LT software (Version 5.0; Verity Software House, Topsham, ME, USA).

### 2.14. 5-Ethynyl-2′-deoxyuridine (EdU) Incorporation Assays

Cell proliferation was assessed using the BeyoClick™ EdU-488 kit (Beyotime, Shanghai, China) according to the manufacturer’s instructions. The treated cells were incubated with 10 µM EdU working solution in complete medium at 37 °C for 2 h. The medium was then removed, and the cells were washed twice with PBS, fixed with 4% paraformaldehyde at room temperature for 15 min, and permeabilized with 0.3% Triton X-100 in PBS for 15 min. After washing with PBS, the cells were incubated with the Click reaction solution (containing the EdU-488 azide) at room temperature for 30 min in the dark, and the nuclei were stained with Hoechst 33342 (Beyotime, Shanghai, China) for 10 min. Fluorescence images were captured using a fluorescence microscope, and the percentage of EdU-positive cells was calculated as the ratio of EdU-positive to total Hoechst-stained cells using ImageJ software (version 5.2.5).

### 2.15. Immunofluorescence Staining

After 5 days of differentiation induction, immunofluorescence staining was performed on the differentiated myoblasts. Cells were fixed with 200 µL per well of 4% paraformaldehyde (Biosharp, Hefei, Anhui, China) for 30 min at room temperature, and washed 3–5 times with PBS. Cells were permeabilized with 0.3% Triton X-100 (Merck KGaA, Darmstadt, Germany) for 30 min at room temperature, washed again, and blocked with 5% bovine serum albumin (Biofroxx, Einhausen, Germany) for 1 h at room temperature. The cells were then incubated overnight at 4 °C with primary antibodies against MyHC isoforms (anti-MYH3, Mouse, Santa Cruz Biotechnology, Dallas, TX, USA; Cat#: sc-53091; anti-MYH7, Rabbit, Abiowell, Changsha, Hunan, China; Cat#: AWA61703; both at 1:250). After washing, cells were incubated with pig-specific fluorophore-conjugated secondary antibodies (Goat Anti-Mouse IgG, Proteintech, Shanghai, China; Cat#: RGAM002 and Goat Anti-Rabbit IgG, Proteintech, Shanghai, China; Cat#: RGAR002, both at 1:500) for 40 min at room temperature in the dark, and nuclei were counterstained with Hoechst 33342 (Beyotime, Shanghai, China; 1:1000) for 10 min. Fluorescence images were acquired under a 10× objective using an inverted epifluorescence microscope (Leica Microsystems, Wetzlar, Germany), with three random fields captured per biological sample. The differentiation index was calculated as the percentage of MyHC-positive cells among total cells, and the fusion index was defined as the proportion of nuclei within myotubes (containing ≥2 nuclei) relative to the total number of nuclei.

### 2.16. Statistical Analysis

GraphPad Prism 9.5.1 (GraphPad Software, San Diego, CA, USA) was used for data analysis and graph plotting. All experiments were conducted with three independent biological replicates. Data are presented as the mean ± standard deviation (SD). The differences between two groups were analyzed with an unpaired *t*-test, and those among multiple groups were analyzed with one-way ANOVA followed by Tukey’s multiple comparison test; * *p* < 0.05, ** *p* < 0.01, *** *p* < 0.001.

## 3. Results

### 3.1. The −143 A>C SNP in the Porcine DLD Promoter Enhances KLF2 Binding to Suppress DLD Transcription

**Figure 1:**
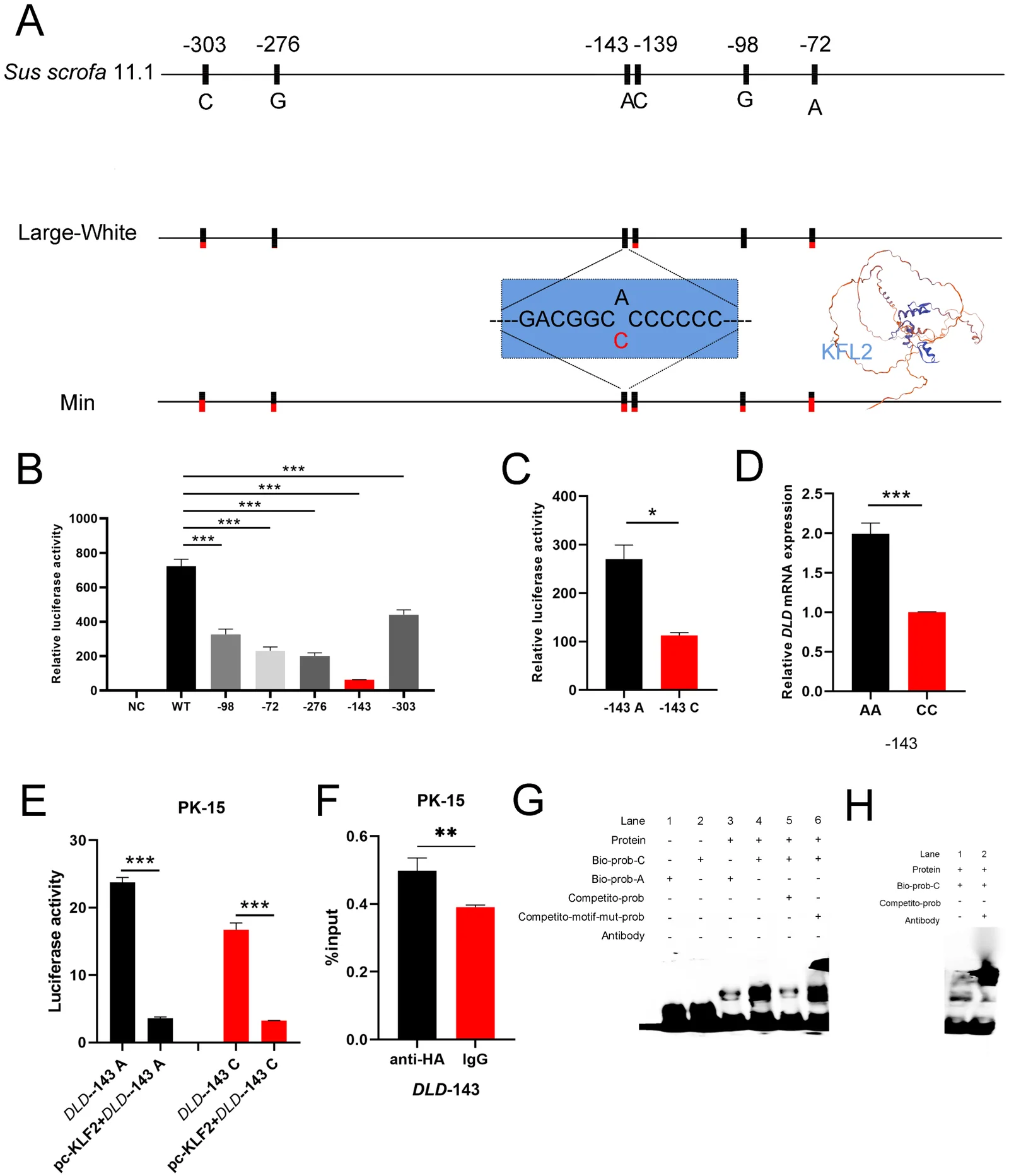
Identification and functional characterization of a regulatory SNP in the porcine *DLD* promoter. (**A**) Schematic of various porcine *DLD* promoter regions, including Sus scrofa 11.1, Large White, and Min pig, six polymorphic sites and a predicted KLF2 binding site at position −143. (**B**) Relative luciferase activity of wild-type (WT: Sus scrofa 11.1) and single-site mutant reporters (−303: C>G, −276: G>T, −143: A>C, −98: G>C, −72: A>G) constructs transiently transfected into PK-15 cells, *n* = 3. (**C**) Dual-luciferase reporter assay comparing the transcriptional activity of the −143 A and C alleles, *n* = 3. (**D**) Relative *DLD* mRNA abundance in the *longissimus dorsi* muscle of pigs with defined genotypes at the −143 locus, *n* = 3. (**E**) Dual-luciferase reporter assay in PK-15 cells co-transfected with KLF2 overexpression or empty vector, together with the −143 A or C allele reporter constructs, *n* = 3. (**F**) Chromatin immunoprecipitation (ChIP) coupled with qPCR, *n* = 3. (**G**) EMSA of biotin-labeled A- and C-allele probes incubated with 293T cellular nuclear extracts, with competition by unlabeled C- or A-allele oligonucleotides, *n* = 3. (**H**) Supershift assay of the C-allele probe using anti-KLF2 antibody, *n* = 3. Data are presented as mean ± SD. * *p* < 0.05, ** *p* < 0.01, *** *p* < 0.001.

Previous studies have revealed significant transcriptional differences in the *DLD* gene across distinct muscle anatomical sites, and this phenomenon is conserved across multiple species [19,20,21]. Given the distinct myofiber composition across skeletal muscle depots, unravelling the regulatory basis of *DLD* expression disparities carries tangible implications for the genetic improvement of pork quality. To interrogate promoter variants of *DLD* as causal regulators of breed-specific transcriptional divergence and meat quality differences, we compared Large White and Min pigs, and compared the sequences against the Sus scrofa 11.1 reference genome. Genotype counts, genotype frequencies, allele frequencies, haplotypes and pairwise linkage disequilibrium between these variants are summarized in Table S2. As shown in Figure 1A, we identified six polymorphic sites at positions −303, −276, −143, −139, −98 and −72 relative to the transcription start site. The −139 locus was excluded from dual-luciferase reporter assays owing to its multiallelic nature. We therefore restricted functional validation of transcriptional activity to the remaining five biallelic variants. To narrow down key functional loci, we generated five separate fluorescent reporter vectors, each carrying a single SNP mutation. Comparison with the wild-type vector demonstrated that all single-site mutations significantly attenuated promoter activity, among which the −143 variant conferred the strongest repressive influence (*p* < 0.001, Figure 1B).

To further validate the regulatory effect of the −143 polymorphic site on *DLD* promoter activity, we performed dual-luciferase reporter assays and in vivo expression quantification, respectively. Dual-luciferase analysis revealed markedly reduced relative luciferase activity for the C allele compared with the A allele (*p* < 0.05, Figure 1C). In parallel, in vivo mRNA quantification demonstrated that individuals carrying the CC genotype exhibited substantially lower relative *DLD* transcript abundance than AA homozygotes at the −143 locus (*p* < 0.001, Figure 1D).

Bioinformatic prediction indicated that the −143 A>C polymorphism resides within a putative binding motif of transcription factor KLF2 (5′-GACGGCCCCCCC-3′) (Figure 1A). To functionally interrogate KLF2-mediated transcriptional regulation of DLD, we co-transfected porcine KLF2 overexpression constructs alongside luciferase reporter vectors carrying the alternative −143 A or C promoter haplotypes in PK-15 cells. As shown in Figure 1E, KLF2 overexpression significantly attenuated the promoter activity of both the A and C alleles (*p* < 0.001). Chromatin immunoprecipitation (ChIP) assays further demonstrated significant enrichment of the *DLD* promoter fragment containing the −143 locus in HA-tagged KLF2 immunoprecipitates compared with IgG controls in PK-15 cells (*p* < 0.01, Figure 1F), corroborating the direct binding of KLF2 to the *DLD* promoter. To determine whether KLF2 directly binds the −143 polymorphic site, we performed electrophoretic mobility shift assays (EMSAs) using biotin-labelled probes. As shown in Figure 1G, a specific protein-DNA complex was prominently detected with the C-allele probe (lane 4), whereas the A-allele probe showed markedly weaker binding (lane 3). The C-allele-specific complex was effectively abolished by an excess of unlabeled C-allele competitor (lane 5) but remained resistant to competition by an unlabeled A-allele competitor (lane 6), confirming sequence-specific binding to the C-allele motif. Furthermore, supershift assays using a KLF2-specific antibody resulted in a retarded migration of the C-allele-protein complex (Figure 1H), confirming that KLF2 specifically binds the −143 C allele of the DLD promoter.

Collectively, these multiple complementary lines of evidence suggest that the C allele at the −143 locus of the porcine *DLD* promoter suppresses transcription presumably via enhanced KLF2-dependent repression, leading to diminished endogenous *DLD* mRNA expression.

### 3.2. The Overexpression of Porcine DLD Promotes Myocyte Proliferation

**Figure 2:**
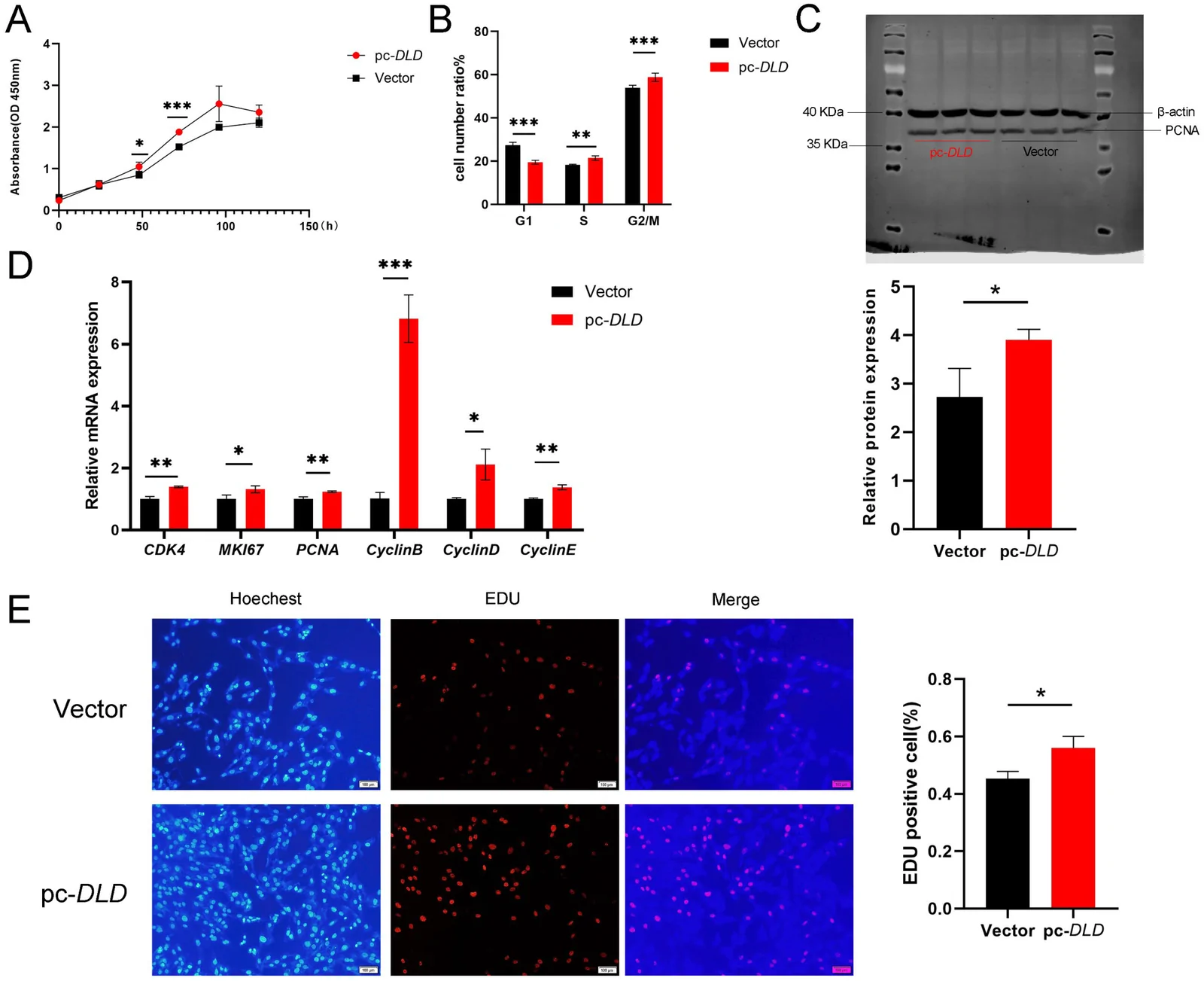
Effect of porcine *DLD* overexpression on myoblast proliferation. (**A**) CCK-8 assay of porcine primary myoblasts transfected with pCMV-HA-*DLD* (pc-*DLD*) or pCMV-HA (empty vector), *n* = 3. (**B**) Flow cytometric analysis of cell cycle distribution in transfected myoblasts, *n* = 3. (**C**) Western blot analysis of PCNA protein levels, *n* = 3. (**D**) Quantitative RT-PCR analysis of cell cycle regulatory genes (*CDK4*, *MKI67*, *PCNA*, *CyclinB*, *CyclinD*, and *CyclinE*), *n* = 3. (**E**) EdU incorporation assay (red) with Hoechst 33342 nuclear counterstain (blue). Scale bar, 100 μm, *n* = 3. Data are presented as mean ± SD. * *p* < 0.05, ** *p* < 0.01, *** *p* < 0.001.

To investigate the relationship between *DLD* gene and skeletal myocyte development in domestic pigs, we overexpressed *DLD* in porcine primary myoblasts using a pCMV-HA-*DLD* construct and monitored cell growth over time. CCK-8 assays revealed that *DLD* overexpression significantly enhanced cell proliferation compared to the empty vector control, with the difference becoming progressively more pronounced from 24 to 72 h post-transfection (*p* < 0.05, Figure 2A). To determine whether the enhanced proliferation was attributable to altered cell cycle dynamics, we performed flow cytometry and found that *DLD*-overexpressing cells exhibited a markedly reduced G1-phase population (19.46% ± 0.53% vs. 27.39% ± 0.76% in control), a concomitant increase in the S-phase fraction (21.44% ± 0.58% vs. 18.31% ± 0.14%), and an elevated G2/M-phase fraction (58.80% ± 1.09% vs. 53.97% ± 0.64%) (*p* < 0.05, Figure 2B, Figure S1A). Moreover, Western blot analysis demonstrated that *DLD* overexpression substantially elevated PCNA levels, a well-established marker of DNA replication and cell proliferation (*p* < 0.05, Figure 2C). Quantitative RT-PCR further revealed that *DLD* overexpression significantly upregulated the transcriptional expression of multiple cell cycle regulators, including *CDK4*, *MKI67*, *PCNA*, *CyclinB*, *CyclinD*, and *CyclinE*, among which *CyclinB* exhibited the most pronounced upregulation (*p* < 0.05, Figure 2D). To visually image the impact of *DLD* on myocyte proliferation, we performed EdU incorporation assays. Fluorescence microscopy showed a higher density of EdU-positive cells in the *DLD*-overexpression group compared to the control (Figure 2E). Quantification confirmed that the proportion of EdU-positive cells was significantly increased upon *DLD* overexpression (*p* < 0.05, Figure 2E). Overall, these findings suggest porcine *DLD* as a positive regulator of myocyte proliferation, which may promote cell cycle progression, specifically facilitating the G2/M transition.

### 3.3. DLD Knockdown Impairs Myoblast Proliferation

**Figure 3:**
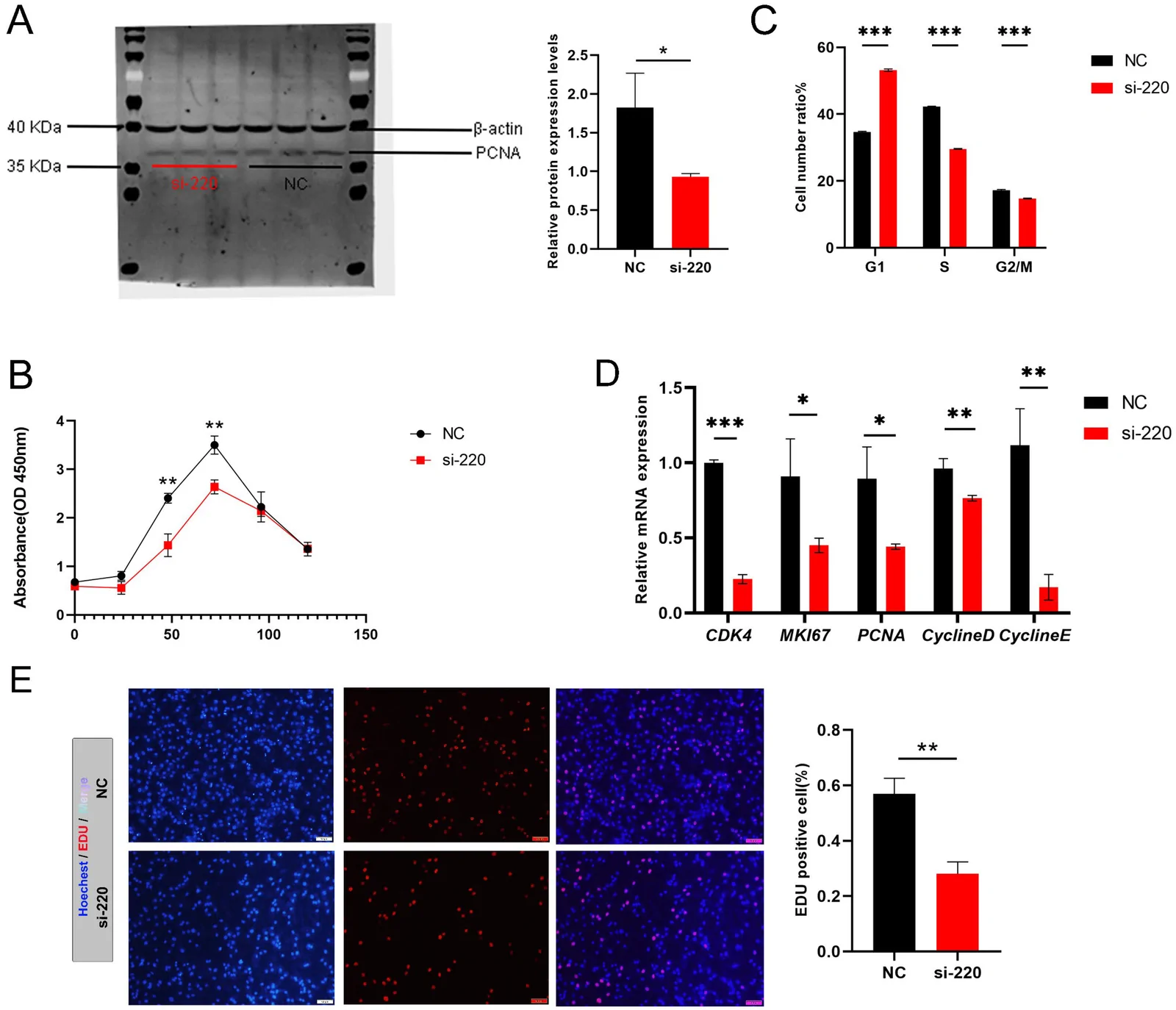
*DLD* regulates myoblast proliferation. (**A**) Western blot analysis of PCNA, *n* = 3. (**B**) CCK-8 assay of porcine primary myoblasts transfected with empty vector (Negative control, NC) or si-*DLD* 220 (si-220), *n* = 3. (**C**) Flow Cytometry, *n* = 3. (**D**) qRT-PCR of cell cycle regulatory genes, *n* = 3. (**E**) EdU incorporation assay in porcine primary myoblasts transfected with si-220 or NC, *n* = 3. Scale bar: 100 μm. Data are presented as mean ± SD. * *p* < 0.05, ** *p* < 0.01, *** *p* < 0.001.

To complement our overexpression studies and establish the necessity of *DLD* for myocyte proliferation, we constructed three independent siRNAs (si-673, si-1498, si-220) and selected si-220 for subsequent experiments based on its knockdown efficiency (Figure S1B). Concomitant with reduced *DLD* expression, PCNA protein levels were markedly decreased (*p* < 0.001, Figure 3A). Consistent with this observation, CCK-8 assays revealed that *DLD* knockdown significantly suppressed cell proliferation compared to the NC treatment, with the inhibitory effect becoming progressively more pronounced from 24 to 72 h post-transfection (*p* < 0.001, Figure 3B). Flow cytometric analysis further demonstrated that *DLD* knockdown induced cell cycle arrest at the G1 phase. The proportion of cells in G1 phase increased from 34.68% ± 0.10% in the NC group to 53.18% ± 0.19% in the si-220 group, accompanied by a decrease in S-phase cells (from 42.28% ± 0.07% to 29.58% ± 0.07%) and G2/M-phase cells (from 17.24% ± 0.13% to 14.74% ± 0.08%) (*p* < 0.001, Figure 3C, Figure S1C). Additionally, we assessed the transcriptional expression of key cell cycle regulatory genes, and the results showed that *DLD* knockdown significantly downregulated the mRNA levels of *CDK4*, *MKI67*, *PCNA*, *Cyclin D*, and *Cyclin E* (*p* < 0.05, Figure 3D). EdU incorporation assays further confirmed that DNA synthesis was substantially impaired following *DLD* knockdown, as evidenced by a ~50% reduction in EdU-positive cells (*p* < 0.001, Figure 3E).

### 3.4. DLD Suppresses Slow-Twitch Myogenic Differentiation in Porcine Primary Myoblasts

**Figure 4:**
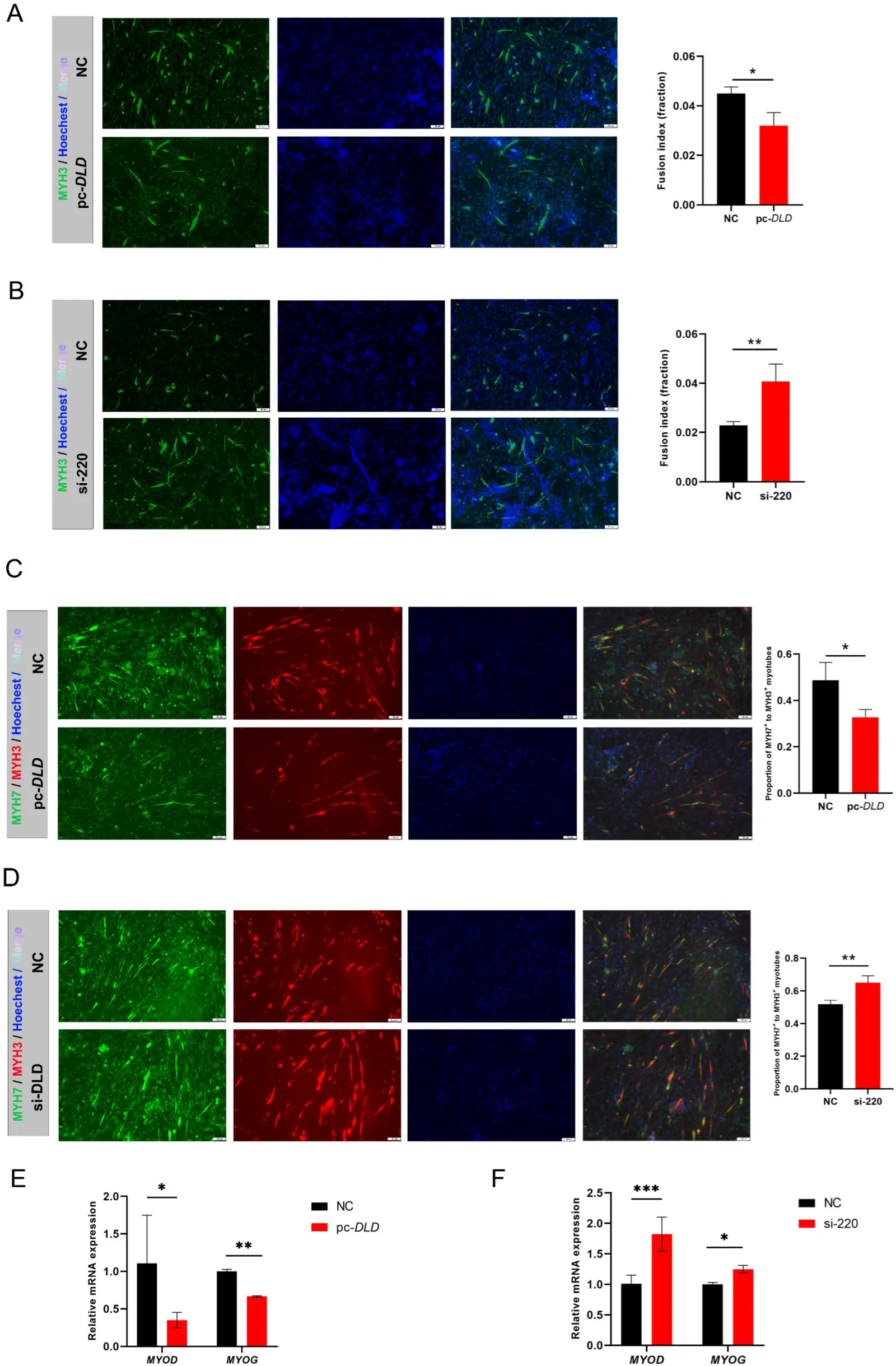
*DLD* inhibits myoblast fate toward a slow-twitch myofiber phenotype. (**A**) Left: Immunofluorescence staining of MYH3 (green) and Hoechst 33342 (blue) in myoblasts transfected with pc-*DLD* or empty vector (NC); Right: quantification of fusion index, *n* = 3. (**B**) Left: Immunofluorescence staining of MYH3 (green) and Hoechst 33342 (blue) in myoblasts transfected with si-220 or NC; Right: quantification of fusion index, *n* = 3. (**C**,**D**) Left: Double immunofluorescence staining of MYH7 (red), MYH3 (green), and Hoechst 33342 (blue) in pc-*DLD* or si-220-transfected myoblasts; Right: Quantification of the ratio of MYH7-positive to MYH3-positive myotubes, *n* = 3. (**E**) qRT-PCR analysis of myogenic regulatory factors *MYOD* and *MYOG* in pc-*DLD*-transfected myoblasts, *n* = 3. (**F**) qRT-PCR analysis of *MYOD* and *MYOG* in si-220-transfected myoblasts, *n* = 3. Scale bar, 100 μm. Data are presented as mean ± SD. * *p* < 0.05, ** *p* < 0.01, *** *p* < 0.001.

Given that *DLD* was originally identified from comparative screening between two anatomically distinct skeletal-muscle sites with divergent myofiber-type compositions [22], we next investigated whether *DLD* governs the differentiation fate of myoblasts. To investigate the role of *DLD* in skeletal muscle fiber type specification, we first established gain- and loss-of-function models in porcine primary myoblasts. Immunofluorescence staining of MYH3 revealed that *DLD* overexpression significantly reduced the fusion index compared with the NC group (*p* < 0.05, Figure 4A). Conversely, si-220 treatment markedly increased fusion index relative to the NC group (*p* < 0.01, Figure 4B). We next examined the effect of *DLD* on slow-twitch myogenic differentiation, using MYH7/MYH3 double immunofluorescence as a readout of slow-twitch commitment in newly formed myotubes. *DLD* overexpression significantly reduced the ratio of MYH7^+^ to MYH3^+^ myotubes compared with the NC group (*p* < 0.05, Figure 4C), suggesting that *DLD* expression suppresses slow-twitch myogenic differentiation. In contrast, *DLD* depletion markedly enhanced the proportion of MYH7^+^ to MYH3^+^ myotubes relative to the NC group (*p* < 0.01, Figure 4D).

To dissect the molecular basis by which *DLD* modulates myofiber subtype switching, we quantified the mRNA expression of core myogenic regulatory factors. *DLD* overexpression significantly suppressed the transcription of *MYOD* and *MYOG* (*p* < 0.05, Figure 4E), which is consistent with our earlier finding that *DLD* restrains overall myogenic differentiation capacity. Conversely, *DLD* knockdown markedly upregulated the expression of *MYOD* and *MYOG* (*p* < 0.05, Figure 4F), further supporting that endogenous *DLD* inhibits myogenic differentiation. Collectively, these findings illustrate that *DLD* restrains terminal myogenic differentiation and suppresses slow-twitch myogenic commitment in porcine myoblasts.

## 4. Discussion

Skeletal muscle maturation is accompanied by a metabolic rewiring in which proliferative myoblasts utilize glycolysis, whereas differentiating cells activate mitochondrial oxidative phosphorylation [23,24,25]. DLDH dysfunction destabilizes mitochondrial metabolism in myocytes, blunts oxidative phosphorylation and energy generation, and ultimately compromises muscle development and regenerative capacity after injury [25,26,27]. In this study, we demonstrate that *DLD* overexpression in porcine myoblasts reduces the G1-phase population, increases the S/G2–M-phase fraction and upregulates proliferative markers including PCNA and cyclin family members, whereas *DLD* knockdown elicits G1-phase arrest and diminished DNA synthesis activity. These observations are consistent with the established role of metabolic enzymes in governing myoblast proliferation [28,29].

However, myoblast proliferation and fiber-type specification represent functionally distinguishable biological processes [30]. Proliferative expansion sets the total number of myofibers, while fiber-type specification shapes myofiber subtype identity and underpins the functional diversity of skeletal muscle and meat quality [31,32,33].

Using combined immunohistochemical, histochemical and in situ hybridization approaches, Lefaucheur et al. systematically characterized porcine myofiber types and confirmed the abundant presence of fast-glycolytic type IIb fibers in the *longissimus dorsi* [34]. Strong directional selection over the 10,000-year history of pig domestication has reshaped the core composition of skeletal muscle fibers, generating significant phenotypic variation between domestic pigs and wild boars [35,36]. Consistent with this evolutionary pattern, histological evidence from Fazarinc et al. (2017) further validates that artificial selection substantially elevates type IIb fiber proportion in domestic pigs relative to wild boars [36], supporting an association between domestication and altered fiber-type composition. Transgenic pig models and multi-omic analyses have further indicated that oxidative soleus muscle exhibited markedly greater mitochondrial content and oxidative pathway protein abundance than glycolytic *longissimus dorsi* [21,37,38,39].

Moreover, divergent selection pressures and breeding priorities applied to distinct populations have stratified breeds into unique myofiber subtypes and meat quality phenotypes [35,40]. Therefore, in this study, we performed a comparative sequence analysis of the *DLD* gene promoter between commercial lean-type breeds (Large White) and the Min pig, a local Chinese breed renowned for its superior meat quality traits. Among six polymorphic sites, the −143 A>C variant emerged as the predominant functional determinant of transcriptional output. Single-site mutagenesis demonstrated that this polymorphism conferred the strongest repressive effect on promoter activity, a finding corroborated by both in vitro luciferase reporter assays and in vivo transcript quantification in animals of defined genotype. Individuals homozygous for the C allele exhibited markedly attenuated *DLD* expression relative to AA homozygotes.

Bioinformatic and experimental evidence converged to identify KLF2 as the trans-acting mediator of this allelic effect. KLF2 is a pivotal transcriptional regulator of myoblast fusion; the canonical extracellular-signal-regulated kinase 5-specificity protein 1-KLF2 signaling axis functions as a secondary differentiation module independent of the MYOD master regulatory network [41]. Despite well-established roles of KLF2 in orchestrating myoblast programs, its direct transcriptional targets among mitochondrial metabolic enzymes remain largely uncharacterized. This gap prompted us to interrogate whether porcine *DLD* carries functional KLF2 binding motifs in its promoter region, and whether natural genetic variants at this site disrupt KLF2-dependent transcriptional control of *DLD* to remodel myogenic fate. In this study, we identified that the C allele strengthened KLF2 DNA-binding affinity and substantially enhanced KLF2-mediated transcriptional repression of *DLD*. Individuals harboring the −143 CC genotype are expected to exhibit reinforced KLF2 binding, which potentiates transcriptional repression and drives lower *DLD* expression, thereby attenuating the inhibitory effect of *DLD* on oxidative myogenic differentiation. Conversely, the AA genotype, through weakened KLF2 binding, relieves transcriptional repression and sustains relatively higher *DLD* expression. These findings suggest that the −143 C allele confers higher KLF2-binding affinity than the A allele and enhances transcriptional repression of *DLD*.

On the other hand, our functional assays revealed that *DLD* overexpression significantly reduced the proportion of MYH7^+^ myotubes and lowered fusion index, indicating that *DLD* suppresses slow-twitch myogenic commitment while restraining terminal differentiation. This observation suggests that *DLD* may antagonize oxidative myogenic programs indirectly by sustaining proliferative capacity, rather than through direct transcriptional activation of fast-twitch markers; meanwhile, this softens the conventional view that mitochondrial oxidative enzymes uniformly promote oxidative/slow-twitch fiber development [42].

The molecular mechanism underlying this differentiation modulation may lie in the intimate connection of *DLD* to pyruvate metabolism and the intracellular acetyl-CoA, which is a substrate not only for the TCA cycle but also for histone acetylation that dictates chromatin accessibility [24,25,26]. *DLD* overexpression may modulate the cellular availability of acetyl-CoA, thereby influencing the epigenetic landscape at myogenic loci and altering the balance between proliferation and differentiation [23,26,43,44,45,46,47]. In parallel, *DLD*-dependent metabolic flux remodeling shifts the cellular NADH/NAD^+^ redox balance, which could in turn tune the catalytic activity of NAD^+^-dependent sirtuin-family deacetylases, including SIRT1 [26,48,49]. However, these interpretations remain speculative and will require further experimental validation in future work.

Several limitations of this study warrant consideration. First, although our data identify the −143 A>C variant as a functional regulator of *DLD* expression, and *DLD* itself as a determinant of myoblast proliferation and slow-twitch differentiation potential, the causal chain linking this SNP to myofiber-type specification in vivo remains indirect. Second, our single-site reporter assays illustrate the regulatory capacity of individual promoter variants, yet these assays alone cannot pinpoint which variant acts as the primary causal site within naturally occurring haplotypes. In addition, the in vivo *DLD* expression analysis was performed using a limited number of animals (*n* = 3 per genotype), which constrains the generalizability of our observations. Consequently, these data do not enable us to confirm a direct association between the −143 A>C variant and breed-specific meat quality traits. Third, our in vitro assessment of fiber type relied on immunostaining for MYH7 and embryonic MYH3, without adult fast-twitch myosin markers (MYH1, MYH2, MYH4), PGC-1α expression, or direct measurements of oxidative metabolism; a more comprehensive characterization of myofiber subtypes will require this expanded marker panel. Addressing these gaps will require differentiation assays in genotyped primary myoblasts, allele-specific editing of the promoter, and population-scale analyses pairing genotype with muscle fiber-type phenotypes. Future investigations with larger animal populations and matched phenotypic datasets will also be essential to further clarify this potential relationship. These efforts will be critical for translating the molecular mechanism described here into strategies for modulating muscle traits in livestock. From an applied perspective, the −143 A>C SNP represents a mechanistically characterized candidate target for future genomic-selection research. Nevertheless, given the pleiotropic role of *DLD* in cell proliferation, differentiation, and metabolic homeostasis, further in vivo studies will be essential to verify whether this variant causally alters muscle-fiber composition and to assess potential interactions with nutritional and environmental factors before practical breeding application.

## 5. Conclusions

In conclusion, this study identifies the −143 A>C polymorphism in the porcine *DLD* promoter as a regulatory variant for myogenic differentiation. The C allele enhances binding of the transcription factor KLF2, thereby intensifying KLF2-mediated transcriptional repression and diminishing basal *DLD* expression. Moreover, *DLD* serves as a positive regulator of myoblast proliferation while acting as a negative regulator of terminal differentiation and slow-twitch myogenic commitment. These findings elucidate a molecular mechanism connecting promoter variation, transcription factor occupancy, and muscle fiber-type determination, and position the −143 A>C SNP as a promising candidate target for genomic selection strategies aimed at optimizing muscle fiber composition and enhancing pork quality.

## Data Availability

The datasets presented in this article are not readily available due to potential intellectual-property considerations for subsequent breeding applications. Requests to access the datasets should be directed to the corresponding author.

## Supplementary Materials

The following supporting information can be downloaded at: https://www.mdpi.com/article/10.3390/ani16213391/s1, Figure S1: Effects of *DLD* overexpression and knockdown on cell-cycle distribution of porcine primary myoblasts; Table S1: Primers, siRNAs, and probes used in this study; Table S2: Genotype and allele frequencies of six variants in the promoter region of *DLD* in Min and Large White pigs; Table S3: Raw data of the myotube fusion assay.
