# Supplementary material for: In Vitro Functional Characterization of the −143 A>C Variant in the Porcine DLD Promoter and Its Modulation of KLF2-Dependent Repression and Myoblast Phenotypes
Source: Animals (Basel). 2026 Oct 27;16(21):3391. doi: 10.3390/ani16213391 (PMC13762358; doi:10.3390/ani16213391)
Supplement: Supplementary file 1 [file animals-16-03391-s001.zip › Figure S1.pdf]

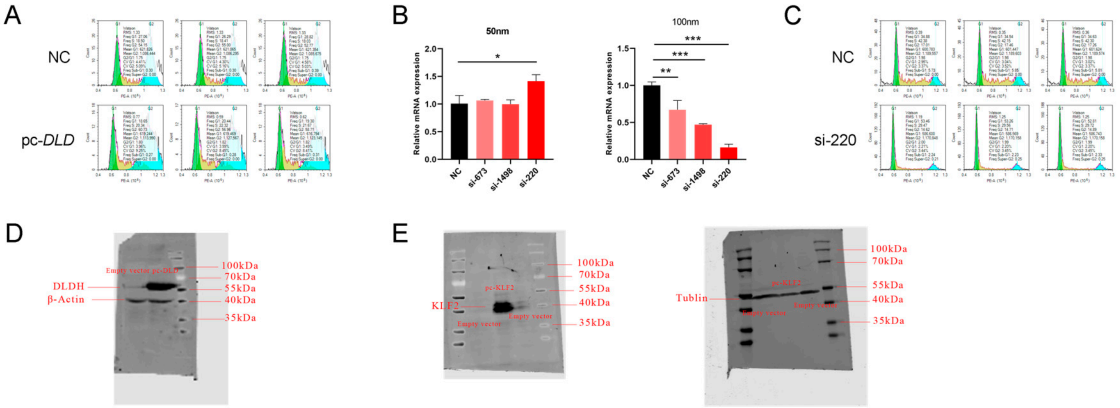


**Supplementary Figure S1.** Effects of *DLD* overexpression and knockdown on cell-cycle distribution of porcine primary myoblasts. (A) Representative flow cytometry histograms and quantitative analysis of cell cycle phases in porcine primary myoblasts transfected with pCMV-HA-*DLD* (pc-*DLD*) or empty vector (NC). (B) Knockdown efficiency of *DLD*-targeting siRNAs assessed by qRT-PCR, n = 3. (C) Representative flow cytometry histograms and quantitative analysis of cell cycle phases in porcine primary myoblasts transfected with DLD-targeted siRNA (si-220) or negative control (NC). (D) Western blot validation of DLDH protein overexpression in cells transfected with pc-DLD or empty vector. β-Actin served as the loading control. (E) Left: Western blot validation of KLF2 protein overexpression in cells transfected with pc-KLF2 or empty vector; Right: Tubulin served as the loading control. Data are presented as mean ± SD. *$P < 0.05$, **$P < 0.01$, ***$P < 0.001$.
